# Supplementary material for: RNA In Situ Hybridization for Detecting Gene Expression Patterns in the Abdomens and Wings of Drosophila Species
Source: Methods Protoc. 2021 Mar 10;4(1):20. doi: 10.3390/mps4010020 (PMC8005959; doi:10.3390/mps4010020)
Supplement: Supplementary file 1 [file mps-04-00020-s001.pdf]

Supplementary Materials

Table S1. List of primers used to prepare probes for ISH.

The *D. guttifera t* exon 5 forward and reverse primer pair was used to amplify *D. guttifera* genomic DNA to produce the probe used to perform the *tan* ISH in *D. palustris* and *D. subpalustris*. The forward and reverse primer pair for *D. guttifera y* exon 2 was used to amplify *D. guttifera* genomic DNA to develop the probe to determine *yellow* gene expression in *D. guttifera* and *D. deflecta*. The *D. palustris* forward and reverse primer pair for *y* exon 2 was used to amplify *D. quinaria* and *D. recens* genomic DNA to make the probes used to determine *yellow* gene expression patterns in both *D. recens* and *D. quinaria.* We used the probes generated from a different species' DNA to perform ISH due to the close evolutionary relationships of species within the *quinaria* species group. All internal forward and internal reverse primer pairs were used for verification of the gene identity during the probe-making process.

**Primer Name**	**Primer Sequence**
D. guttifera t exon 5 forward	CAGCGTCTGCTTGGCCACACG
D. guttifera t exon 5 reverse	TTGCCGCTGCGCAACAATTCGG
D. guttifera t exon 5 internal forward	GCTGAATCATTACTACTTTGTGG
D. guttifera t exon 5 internal reverse	AATGGTGTTGATGCTGAACACG
D. palustris y exon 2 forward	GAGGAGGGCATCTTTGGC
D. palustris y exon 2 reverse	CGATGCCATGGAATTGCGG
D. palustris y exon 2 internal forward	TCTCGCACCGAGGACAGC
D. palustris y exon 2 internal reverse	CGATCAGATTGAACAGCTCG
D. melanogaster wg exon 4 forward	CACGTCCAAGCGGAGATGCG
D. melanogaster wg exon 4 reverse	GGCGACGGCATGTTCGGGTG
D. melanogaster wg exon 4 internal forward	TGCCATGGCATGTCCGGATCG
D. melanogaster wg exon 4 internal reverse	GTTCAGCATACGCTCCTCCTCC
pGEM®-T Easy M13F	GTAAAACGACGGCCAGT
pGEM®-T Easy M13R	CAGGAAACAGCTATGAC

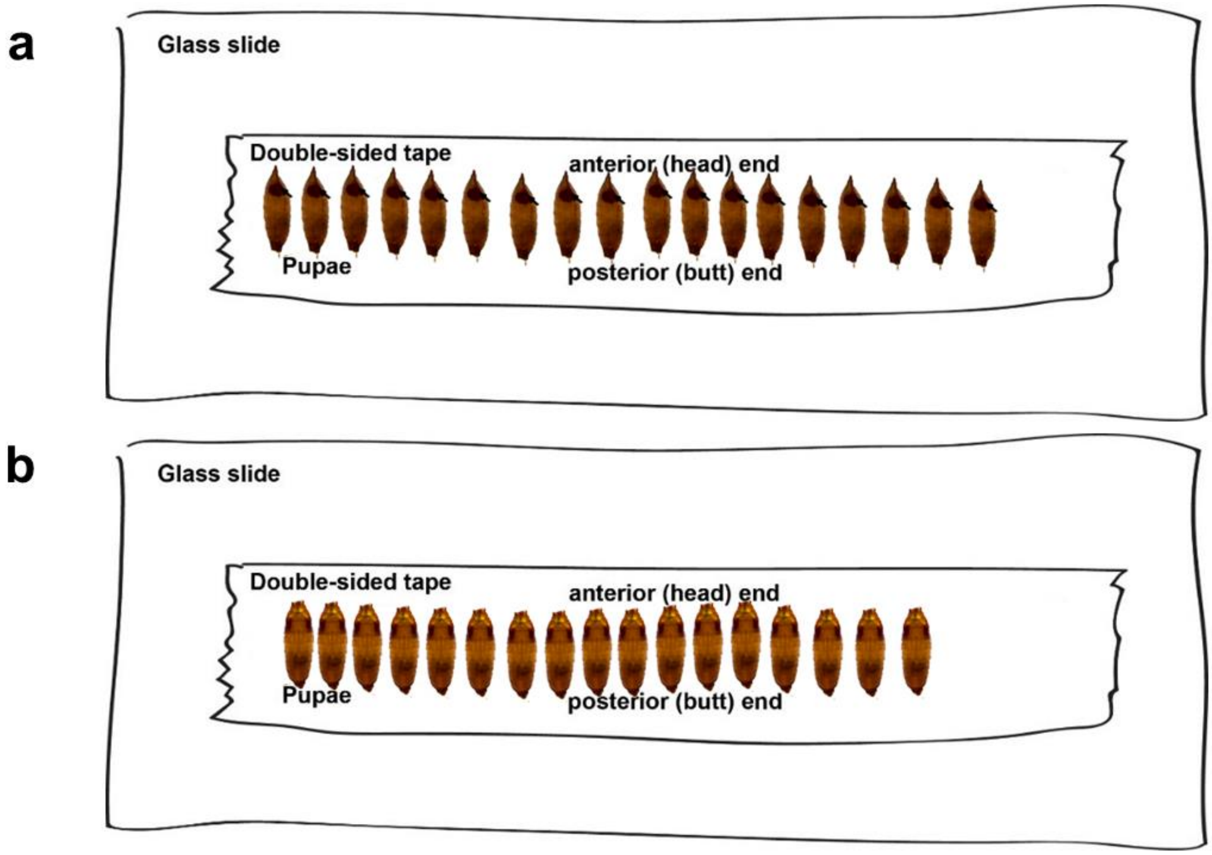

Figure S1. *D. guttifera* pupae lined up for lateral and dorsal cuts. (**a**) The pupae were positioned on the side to perform a cut that separates the dorsal from the ventral half (lateral cut) (**b**) the pupae were placed with their ventral side facing the tape to make a cut between both eyes (dorsal cut).

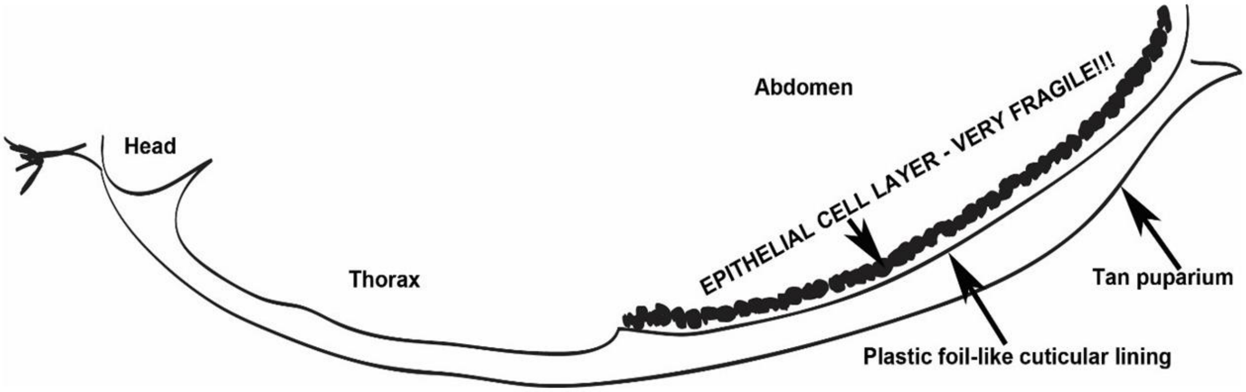

Figure S2. A sketch of a *Drosophila* pupal abdomen showing the internal epithelial cell layer and the cuticular lining holding the cells.

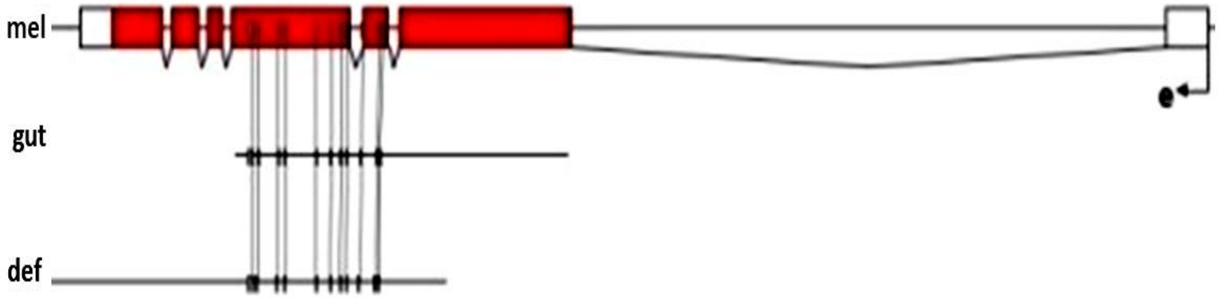

Figure S3. A graphical representation of the conserved region on the *ebony* gene of *D. melanogaster* (top row), *D. guttifera* (gut), *and D. deflecta* (def) [24].
